# Enhancement of Liver Targetability through Statistical Optimization and Surface Modification of Biodegradable Nanocapsules Loaded with Lamivudine

**DOI:** 10.1155/2023/8902963

**Published:** 2023-11-18

**Authors:** Srikar Grandhi, Moawia Al-Tabakha, Prameela Rani Avula

**Affiliations:** ^1^Department of Pharmaceutical Sciences, Vignan's Foundation for Science Technology and Research, Vadlamudi, Guntur 522213, India; ^2^Department of Pharmaceutical Sciences, College of Pharmacy and Health Sciences, Ajman University, P.O. Box 346, Ajman, UAE; ^3^Centre of Medical and Bio-Allied Health Sciences Research Centre, Ajman University, P.O. Box 346, Ajman, UAE; ^4^University College of Pharmaceutical Sciences, Acharya Nagarjuna University, Nagarjuna Nagar, Guntur 522510, India

## Abstract

The intention of the current work was to develop and optimize the formulation of biodegradable polymeric nanocapsules for lamivudine (LMV) in order to obtain desired physical characteristics so as to have improved liver targetability. Nanocapsules were prepared in this study as aqueous-core nanocapsules (ACNs) with poly(lactide-co-glycolide) using a modified multiple emulsion technique. LMV was taken as a model drug to investigate the potential of ACNs developed in this work in achieving the liver targetability. Three formulations factors were chosen and 3^3^ factorial design was adopted. The selected formulation factors were optimized statistically so as to have the anticipated characteristics of the ACNs viz. maximum entrapment efficiency, minimum particle size, and less drug release rate constant. The optimized LMV-ACNs were found to have 71.54 ± 1.93% of entrapment efficiency and 288.36 ± 2.53 nm of particle size with zeta potential of −24.7 ± 1.2 mV and 0.095 ± 0.006 h^−1^ of release rate constant. This optimized formulation was subjected to surface modification by treating with sodium lauryl sulphate (SLS), which increased the zeta potential to a maximum of −41.6 ± 1.3 mV at a 6 mM concentration of SLS. The results of *in vivo* pharmacokinetics from blood and liver tissues indicated that hepatic bioavailability of LMV was increased from 13.78 ± 3.48 *μ*g/mL *∗* h for LMV solution to 32.94 ± 5.12 *μ*g/mL *∗* h for the optimized LMV-ACNs and to 54.91 ± 6.68 *μ*g/mL *∗* h for the surface-modified LMV-ACNs.

## 1. Introduction

Lamivudine (LMV) is an antiviral drug of class nucleoside reverse transcriptase inhibitors, used in the hepatitis B treatment. Hepatocytes are the major target for the liver-tropic viruses such as hepatitis B virus (HBV), and these cells are the site of replication for HBV [1, 2]. Hence, any anti-HBV drug like LMV needs to be developed into a dosage form that can deliver the drug predominantly to the liver tissue so as to achieve the highest therapeutic benefit with fewer side effects.

Colloidal drug delivery systems are capable of delivering the loaded drug at the target site of action, thus improving the therapeutic efficacy and reducing the side effects of the drug [3]. Polymeric nanoparticles are physically more stable and flexible for modification. These can be modified into having a wide range of surface properties among the different systems viz. solid lipid nanoparticles, niosomes, and liposomes [4, 5]. Biodegradable polymers like poly (lactide-co-glycolide) (PLGA) are less toxic and digestible in the body fluids. Hence, these are most widely employed currently for nanoparticle preparation [6]. Liver targeting can be achieved by broadly two types of approaches viz. active targeting and passive targeting approaches. Active targeting approaches need liver-specific ligands on the nanoparticle surface otherwise targeting cannot be achieved. Targeting ligands for cell-specific receptors on the liver have to be chosen selectively to guide active targeting of the nanoparticles. Few such receptors on the liver for example are asialoglycoprotein receptor (ASGP-R) on the hepatocytes, galactose receptors on the Kupffer cells, uroplasminogen receptors on the hepatic stellate cells, hyaluronan fibronectin, and denatured collagen receptors on the sinusoidal endothelial cells. Though few such ligands such as lactobionic acid, glycyrrhetinic acid, and asialofetuin are identified and reported, still their reliability and reproducibility are yet to be studied [7]. Targeting by external triggering (like applying an external magnetic field or sound waves) demands sophisticated facility, and also, the patient is needed to be hospitalized [8–10]. Targeted drug delivery by biochemical triggering/impulses may also be possible, but this limits the selection of carrier systems or polymers Taking into account these difficulties and limitations, liver targeting through passive targeting approaches can possibly be simple yet reliable.

Passive targeting of the liver can be achieved by making use of the characteristics of the liver, particularly its endocytosis property. The fate of nanoparticles upon intravenous administration largely depends on their physical characteristics. Those characteristics include particle size and surface properties such as hydrophobicity and zeta potential [11]. Nanoparticles with particle sizes below 200 nm can escape phagocytosis by the reticuloendothelial system (RES)/macrophages and can have high circulatory time in the blood. On the other hand, nanoparticles of size just above 200 nm with a hydrophobic surface and high negative zeta potential are readily phagocytized by RES and delivered into RES-rich organs like the liver [12, 13]. Modification of surface hydrophobicity, charge, and particle size can be achieved by carefully controlling the formulation of nanoparticles. Hence, in this work, passive targeting of the liver through modification of size and surface properties was opted.

The major aim of this work was to develop aqueous-core nanocapsules (ACNs) to achieve high drug entrapment as well as liver targetability of the loaded drug. Few literature reports suggested that ACNs, a novel form of polymeric nanoparticles, have high potential in loading hydrophilic drugs to a greater extent. Vignaud et al. developed ACNs for a high water-soluble drug, doxorubicin HCl. Through these ACNs, the authors could obtain the entrapment efficiency of the doxorubicin HCl to a maximum of 80% [14]. Cosco et al. developed ACNs with PLA for loading a high water-soluble drug, gemcitabine HCL. These authors also reported that the ACNs were efficient in achieving higher loading of water-soluble drugs [15]. Deng et al. also described the efficiency of polymeric ACNs in improving the loading of high water-soluble drugs in their review [16]. LMV, considering its physicochemical properties and therapeutic action in the liver tissue, was taken as a model drug to study the ability of the ACNs to deliver the drug to the liver tissue. The ACNs for LMV were prepared through the W/O/W emulsification method using PLGA RG503H, a hydrophobic and biodegradable polymer. The development of ACNs was performed through the design of experiments (DoE) approach. DoE is a statistical tool whose application is now mandated by the regulatory bodies in pharmaceutical/biotechnological industries. Under the DoE, the preparation of ACNs was designed as a 3^3^ factorial design of response surface methodology. Three formulation parameters were taken as the critical material attributes (CMAs) viz. concentrations of the PLGA and the surfactant in secondary emulsion, and also the nature of the external water phase. The prepared ACNs were subjected to thermal analysis to know the physical state of LMV in the ACNs. Particle size (PS) and surface charge were analyzed by Zetasizer. Morphology of the surface of the ACNs was investigated by transmission electron microscopy (TEM). Also, entrapment efficiency (EE) and *in vitro* drug release studies by the dialysis bag method were performed. To optimize the formulation of ACNs, the responses taken were EE, PS, and drug release rate constant (*k*). Formulation of the ACNs was optimized by the desirability functions approach, a statistical approach, with the target of reduced PS (to have more diffusivity into tissues including the target liver tissue), high EE (to reduce the weight of formulation per dose), and less drug release rate constant (to get a prolonged duration of action). Later, the optimized formulation of LMV-ACNs was subjected to surface modification upon treatment with sodium lauryl sulphate, an anionic surfactant to enhance the negative zeta potential of LMV-ACNs. These formulations were studied for *in vivo* pharmacokinetic studies in rat models to know the hepatic distribution and hepatic bioavailability of the optimized and then surface-modified LMV-ACNs. The final formulation was also studied for its cytotoxicity. Similar work was reported by Srikar and Rani [17] by taking tenofovir as the model drug to develop ACNs with the objective of only formulation optimization. However, in the current work, the optimized formulation of the LMV-ACNs was subjected to *in vivo* pharmacokinetic studies so as to justify its liver targeting potential.

## 2. Materials and Methods

### 2.1. Materials

LMV was obtained from Hetero Drugs Ltd (Hyderabad, India); PLGA RG503H (RESOMER-RG503H) was procured from Sigma-Aldrich (Mumbai, India); MTT [3-(4,5-dimethylthiazol-2-yl)-2,5-diphenyl tetrazolium bromide], trypsin, DMEM (Dulbecco's modified Eagles medium), and phosphate-buffered saline (PBS) were acquired from Sigma Chemicals Co. (St. Louis, MO); fetal bovine serum (FBS) was procured from Gibco; Pluronic F-68, sodium lauryl sulphate (SLS), and Span 80 were acquired from S.D. Fine Chemicals (Mumbai, India). All remaining materials employed in this study were of analytical grade.

### 2.2. Fourier Transform Infrared Spectroscopy (FTIR)

The compatibility of LMV with PLGA, Pluronic F-68, and SLS was tested using FTIR (Alpha, Bruker). Pure LMV and physical mixtures (100 mg of LMV with 100 mg of each of the above excipients) were prepared as pellets in a hydraulic press after thoroughly mixing with potassium bromide. These pellets were individually exposed to scanning in wavelength regions from 4000 cm^−1^ to 400 cm^−1^ [18], and spectra were recorded.

### 2.3. Preparation of LMV-ACNs

#### 2.3.1. Experimental Design

In the present work, LMV-ACNs were developed by modified multiple emulsification (W/O/W). The three most significant formulation parameters were selected based on the knowledge from previous literature, and each was taken at three levels. The ranges of the independent variables were taken based on several preliminary trials. These trials were performed with varying levels of independent factors. The levels for optimization were finalized as the minimum and maximum levels at which nanoparticles were yielded with sufficient suitability to perform characterization studies. These were concentration of polymer out of total weight of nanocapsules (factor A: PLGA RG503H–50%, 62.5% and 75% w/w), the concentration of Pluronic F-68 in the outer aqueous phase (factor B: 0%, 0.25% and 0.5% w/v), and the proportion of glycerol in the outermost water phase (factor C: 0%, 25% and 50% v/v). With Design-Expert v8.0 software, a 3^3^full-factorial design was created for the experiment. There are a total of 28 runs because each combination of the factor levels was treated as a single block with a single center point. The PS, EE, and *k* were chosen as response variables.  Table 1 provides a description of the components and the matching levels that were taken, and Table 2 provides information on the experimental runs.

#### 2.3.2. Preparation of LMV-ACNs

Multiple emulsification method as reported by Cruz et al.[19] with some modifications was employed to develop LMV-ACNs. The same method is employed for the development of the ACNs of tenofovir in our earlier work [17] was employed here with the change of drug into LMV. The inner aqueous phase (W1) was made by solubilizing LMV in the solvent mixture of methanol and water taken at a 1 : 4 ratio. The polymer was solubilized in chloroform to constitute the organic phase (O). Two mL of the W1 was added drop by drop into 10 mL of the O, which was maintained kept mixing at 12000 rpm and continued for 30 minutes to develop the primary emulsion of w/o type. Then, immediately, this emulsion dropped slowly into 20 ml of external phase (glycerol and Pluronic F-68 concentrations were as shown in the table) (W2) under continuous mixing to yield multiple emulsions of W1/O/W2. Mixing was continued until chloroform from the middle organic phase was evaporated to yield PLGA nanocapsules with an aqueous core containing LMV (hence, named LMV-ACNs). Then, the LMV-ACNs were recovered as a pellet from the nanosuspension by centrifugation at 8,000 rpm and 4°C for 30 min (Sorvall ST 8R, ThermoFisher Scientific). The pellet of LMV-ACNs was dispersed in fresh distilled water containing mannitol as cryoprotectant and lyophilized (FDB-5502, Operon) for 24 hours to obtain powdered LMV-ACNs.

#### 2.3.3. Surface Modification

The optimized formulation of LMV-ACNs was subjected to surface modification with the objective of enhancing the negative zeta potential. For this, a sequence of aqueous solutions of SLS was prepared in order to obtain 0.5, 1.0, 2.0, 4.0, 6.0, and 8.0 mM concentrations. The optimized formulation of LMV-ACNs of weight 0.2 g was added separately into 10 mL of each of the above SLS solutions. These mixtures were mechanically stirred (RQ-5 Plus, Remi) at 1000 rpm for about two hours at room temperature and then studied for their zeta potential [20, 21].

### 2.4. Differential Scanning Calorimetry (DSC)

By using the DSC (DSC-60, Shimadzu) on the pure drug, pure polymer, and optimized LMV-ACNs, the physical states of LMV and PLGA in the manufactured ACNs were examined [22]. The samples were prepared as per the process reported [17], and the DSC was carried out by increasing the temperature in a nitrogen atmosphere from 20 to 250 degrees Celsius at a speed of 10°C/min. The spectra were then recorded.

### 2.5. TEM Analysis

The surface of the LMV-ACNs was examined employing a transmission electron microscope (Tecnai G2-30, FEI, Netherlands). The optimized formulation of LMV-ACNs was dispersed in water to sufficient dilution, and a drop of it was attached to the carbonated copper grid and waited till dried. Then, this was viewed under the microscope, and photomicrographs were taken [23].

### 2.6. Particle Size and Zeta Potential

Based on the dynamic light scattering (DLS) concept, ZetaSizer Nano ZS90 (Malvern Instruments, UK) [24] evaluated the sizes and zeta potentials of the produced LMV-ACNs. The analysis was carried out with a fixed scattering angle of 90° and a temperature of 25°C. After properly diluting each sample with distilled water, measurements were made in triplicate for each.

### 2.7. Entrapment Efficiency

After preparation, the obtained LMV-ACNs nanosuspension was centrifuged at 8,000 rpm and 4°C for a period of 30 min. The supernatant was collected and subjected to a spectrophotometry (Evolution 201, ThermoFisher Scientific) assay to estimate the amount of LMV that remained unentrapped into ACNs at a maximum wavelength (*λ*_max_) of 271 nm. From this value, the LMV entrapped can be obtained by subtracting the obtained unentrapped amount from the initial amount of LMV taken [14, 25]. The EE was quantified by using the following formula:

(1) $$\begin{array}{c} \text{EE}\left(\%\right)=\frac{\text{Amount of LMV taken}-\text{ Amount of unentrapped LMV}}{\text{Amount of LMV taken}}\times 100. \end{array}$$

### 2.8. *In Vitro* Drug Release Studies

Drug release for LMV-ACNs was performed with the help of a dialysis bag (Dialysis Membrane-110; HiMedia Lab. Pvt. Ltd., Mumbai) [26, 27]. One dose equivalent LMV-ACNs were dispersed in a small amount of water and transferred into the dialysis bag. 100 mL of 0.1N HCl as buffer medium was taken in a beaker and was kept for continuous stirring at 100 rpm on a magnetic stirrer (1MLH, Remi). The dialysis bag containing the ACNs was immersed in the beaker containing the medium. Samples of two mL from the medium were taken at prefixed time points for a total period of 24 h. After every sampling, two mL of fresh buffer was substituted into the beaker. The samples were quantified by measuring absorbance at 271 nm in a spectrophotometer to quantify the amount of LMV released from the ACNs.

### 2.9. Design Validation and Optimization

Design-Expert software was used to perform the DoE validation. All the formulations of LMV-ACNs, obtained by performing the runs according to the model, were estimated for the selected response variables. The obtained values of these response variables were analyzed statistically by the response surface polynomial quadratic model. Plots of predicted versus actual values were plotted, and for each response, an analysis of variance (ANOVA) was run to see whether the chosen model and design were significant enough to warrant optimization.

To meet the objectives for each of the three responses of the LMV-ACNs, the optimization of the design's chosen formulation elements was carried out. The goals were set as reduced PS (to have more diffusivity into tissues, including the target liver tissue), high EE (to reduce the weight of formulation per dose), and less *k* (to get a prolonged duration of action) [28, 29].

### 2.10. *In Vitro* Cytotoxicity Studies

The MTT test was used to assess the toxicity of LMV-ACNs on HeLa cell lines that were procured from NCCS, Pune [30]. This test was carried out similarly as we reported earlier [17].

### 2.11. *In Vivo* Pharmacokinetic Studies

Male Wistar rats having 236−261 g of body weight were chosen for the *in vivo* biodistribution and pharmacokinetic investigations. The rats were maintained in an animal house at 22 ± 0.5°C temperature with 50 ± 5% RH. The study protocol was studied and accepted by the Institute Animal Ethics Committee (IAEC) of the University College of Pharmaceutical Sciences, Acharya Nagarjuna University, Guntur (IAEC No.: ANUCPS/IAEC/AH/P/20/2015). The rats were maintained for overnight fasting with allowance to take water only until four hours after dosing.

All 24 animals were separated into four groups, containing six animals in every group. The groups were labelled as L1: control; L2: aqueous LMV solution; L3: optimized LMV-ACNs; and L4: surface-modified LMV-ACNs. Except for the control group, all the rats in the remaining three groups were administered with the respective formulation at the same LMV equivalent dose of 7.6 mg/kg [31]. The dose was adjusted to 0.4 mL and was given through the saphenous vein of one leg. Blood samples were taken from the lateral saphenous vein of the second leg at 0.5, 1.0, 2.0, 4.0, 8.0, 12.0, 18.0, and 24.0 h after dosing. After every time point, three animals from each group were sacrificed and the liver was isolated. The animals were exposed to CO_2_ for anesthetization. Then, the anesthetized rats were euthanized carefully by cervical dislocation [32]. The isolated liver was homogenized in an isotonic phosphate buffer of pH 7.4. The tissue was transferred into 10 mL of the buffer in a glass homogenizing cup supplied with the glass-teflon tissue homogenizer (Remi, RQ-127 A/D). The tissue was homogenized for 2 min. at 8000 rpm [33]. Then, the obtained homogenate was subjected to LMV extraction.

#### 2.11.1. Preparation of Biological Samples

Liquid-liquid extraction technique was employed to extract the LMV from the biological samples [34]. Plasma was taken by centrifuging the blood samples at 8000 rpm for 15 min. at 4°C. 100 *μ*L of the plasma sample or liver homogenate, 10 *μ*L of IS solution (Nelfinavir 50 *μ*g/mL) were mixed in a vortex mixer (CM-101 Plus, Remi) for 20 sec., and 1.5 mL of acetonitrile (ACN) was added and again mixed for 15 min. The supernatant was separated and dried on a constant temperature water bath until the complete evaporation of ACN. The dried residue was diluted with the mobile phase. These samples were stored at −25°C until analysis using high-performance liquid chromatography (HPLC).

#### 2.11.2. Sample Analysis

LMV in the biological samples was estimated by a modified and validated HPLC (Infinity II LC System, Agilent) method reported by AV Singh et al. Nelfinavir at 50 *μ*g/mL was added as the internal standard (IS). The mobile phase was composed of 0.25% triethylamine buffer (pH 3.0) and ACN at a 70 : 30 ratio with a flow rate of 1 mL/min. 20 *μ*L of the sample was administered into the column (Poroshell 120 EC-C 18; 4.6 × 100 mm) and ran the system for 5 min. The LMV was detected at 258 nm using a PDA detector.

### 2.12. Statistical Analysis

All the statistical analysis including the ANOVA was performed using the Design-Expert software. The statistical significance was conveyed at *p* < 0.05. All the experimental results were presented as mean ± standard deviation of the three observations.

## 3. Results and Discussion

### 3.1. FT-IR

**Figure 1:**
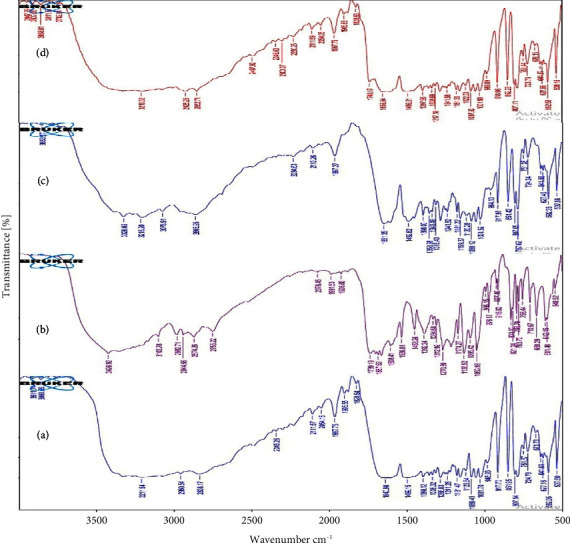
FT-IR spectra of (a) LMV, (b) physical mixture of LMV and PLGA RG503H, (c) physical mixture of LMV and pluronic F-68, and (d) physical mixture of LMV and Span 80.

The spectra of pure LMV and its physical mixtures with the selected excipients are shown in Figure 1. Figure 1(a) of pure LMV exhibited peaks at 3326.53, 1652.46, 1286.25, and 1159.79 cm^−1^ corresponding to the characteristic groups of LMV viz. amino group stretch, carbonyl of cysteine ring, asymmetrical oxathiolane C-O-C stretching, and symmetrical oxathiolane C-O-C stretching, respectively [35]. Spectra (Figures 1(b)–1(d)) of the LMV mixtures with the taken polymer and surfactants also exhibited the above characteristic peaks at the matching wave numbers as those of pure LMV. Hence, there was no incompatibility aroused between LMV and the excipients, and these excipients could be used in the development of formulations for LMV.

### 3.2. TEM

**Figure 2:**
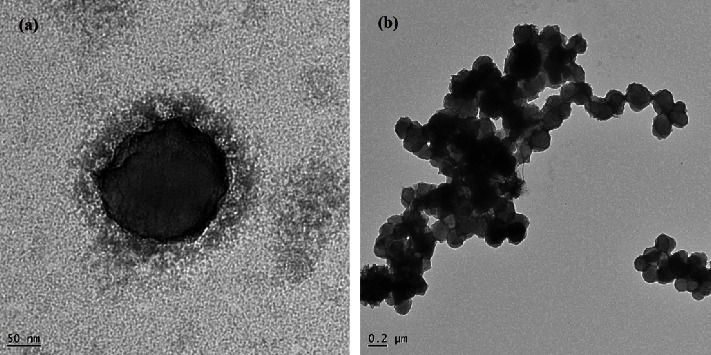
TEM images of optimized formulation of LMV-ACNs prepared from PLGA RG503H.

The surface morphology of the LMV-ACNs was studied by TEM, and the photographs are presented in Figure 2. These illustrated that the prepared ACNs were almost spherical and their surface was smooth and uniform without any dents or protrusions.

### 3.3. DSC

**Figure 3:**
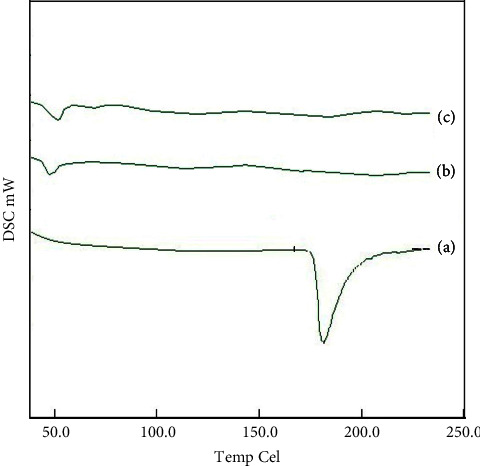
DSC spectra of (a) pure LMV, (b) PLGA RG503H, and (c) LMV-PLGA RG503H ACNs.

During the preparation of the ACNs, LMV was taken as an aqueous solution. Hence, the state of the LMV in the developed ACNs had to be investigated. For this purpose, DSC was performed for the pure LMV, pure PLGA, and the LMV-ACNs, and the obtained spectra are illustrated in Figure 3. The pure LMV spectrum showed an endotherm sharply near 180°C which corresponded to the melting point of LMV, and this confirmed that the pure LMV was in the crystalline state. However, the spectrum of the LMV-ACNs did not indicate any such endotherm. This result designated that the crystalline LMV might be either in the molecular dispersion form or converted into an amorphous form in the ACNs [36]. This could be attributed to the way of incorporating LMV into the nanocapsules during preparation.

### 3.4. Experimental Design

An analysis of the impact of the chosen factors on the response variables was planned to investigate through a 3^3^ factorial design. In order to clarify the quadratic impacts of the factors on the responses, full-factorial designs are helpful. A polynomial quadratic model was used to statistically analyze the responses.

The resulting equations for the responses were

(2) $$\begin{array}{c} \text{EE}=+57.52+7.55*A+2.82*B+12.65*C-1.15*\text{AB}+1.49*\text{AC}+0.57*\text{BC}-5.80*A^{2}-6.44*B^{2}-0.41*C^{2},\\ \text{Particle size}=+315.09+21.17*A-14.23*B-12.39*C+0.38*\text{AB}+5.43*\text{AC}-1.12*\text{BC}-14.35*A^{2}+1.75*B^{2}-9.15*C^{2},\\ k=+0.17-0.028*A-0.018*B-0.028*C+0.0053*\text{AB}-0.0005*\text{AC}-0.00017*\text{BC}+0.0046*A^{2}-0.0022*B^{2}-0.007*C^{2}. \end{array}$$

From the equations, it was deduced that all three of the selected factors had a positive effect on EE; factor A had a positive effect, while factors *B* and *C* had a negative effect on the PS; it was deduced that all three of the factors had a negative effect on *k*. The parts that follow go into further detail about these influences.

### 3.5. Entrapment Efficiency

**Figure 4:**
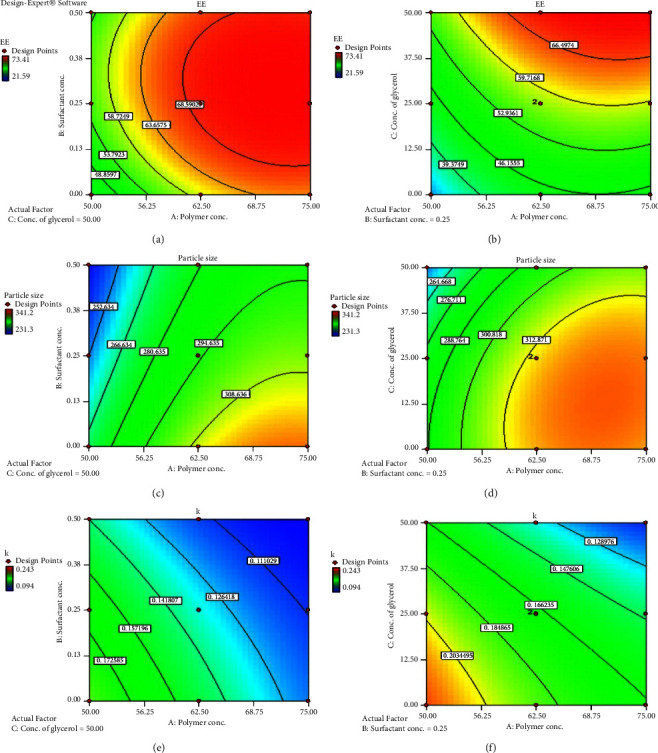
Contour plots showing (a) effect of the factors A and B on entrapment efficiency, (b) effect of the factors A and C on entrapment efficiency, (c) effect of the factors A and B on particle size, (d) effect of the factors A and C on particle size, (e) effect of the factors A and B on drug release rate constant, and (f) effect of the factors A and C on drug release rate constant. (^*∗*^The data that were displayed were the average of three observations, with a statistical significance threshold of *p* < 0.05).

The EE values were obtained in the range of 21.59–73.41% (as shown in Table 2). The influences of the factors on EE are shown in Figures 4(a) and 4(b) for LMV-ACNs in the form of contour plots.

It was very visible how the concentration of the polymer affected the EE, which rose with the level of factor A. This could be as a result of the drug being bound more firmly by a high concentration of polymer, which would reduce drug leakage. Increases in viscosity brought about by increased polymer concentrations also reduced drug diffusion from ACNs, increasing EE [37]. Additionally, at greater polymer levels, the reduction in surface area and increase in path length caused by the larger particles limited the drug's escape through diffusion out of the nanocapsules, increasing the EE [38]. The effect of factor B was intriguing since the EE rose when it was increased from 0.0% to 0.25%, but it decreased when it was increased to 0.5%. The first increase might have resulted from the surfactant stabilizing the emulsion and preventing the drug leakage. However, at a further rise in surfactant concentration, the drug would diffuse out of the nanocapsules and become micellarly soluble in the external aqueous phase [39]. The rise in viscosity and density of the exterior phase of the secondary emulsion upon raising the level of factor C may be responsible for the increase in EE that follows an increase in glycerol content. According to the Stokes–Einstein equation [40], viscosity can decrease diffusion, which means that less amount of drug would leak into the external phase and boost EE.  Table 3 displays the significant results for each of the three factors at *p* < 0.05.

It was discovered that the highest EE was just 74.31%. This may be because the LMV has a high water solubility, which could lead to some leakage. However, in the case of LMV and other comparable highly water-soluble drugs, these EE values for the ACNs produced with PLGA were found to be higher than those reported by other authors using alternative methodologies [41–43]. This shows that the water-soluble drug could be effectively loaded into nanocapsules using both the PLGA polymer and the ACNs approach used in this work.

### 3.6. Particle Size

All formulations of LMV-ACNs were found to have PS ranging from 231.3 to 341.2 nm (Table 2). Contour plots illustrating the impact of the factors on PS are displayed in Figures 4(c) and 4(d). PS increased as the level of factor A increased. This might be as a result of the polymer depositing on the core material's surface after the solvent is removed [44]. Higher polymer levels may, therefore, cause more polymers to settle around the globules in the dispersed phase, resulting in larger particles. At higher viscosities, shearing effectiveness may also be reduced, potentially resulting in the creation of big particles [45]. It was discovered that the surfactant-containing nanoparticles had smaller particles than the surfactant-free ones. This could be as a result of surfactant's capacity to reduce interfacial free energy, which could lead to the formation of a more stable finer emulsion. These findings followed a similar pattern to those reported by Krishnamachari et al.[46] and Gupta et al. [47]. When water was used as the secondary emulsion's external phase, higher particle sizes were seen than when aqueous glycerol was used at 25% and 50% v/v. The glycerol concentration-induced increase in the outer phase's viscosity would prevent the globules from the primary emulsion from aggregating. As can be seen in Table 3, every component was determined to have a significant impact on particle size at *p* < 0.05.

### 3.7. Drug Release Studies

Figures 4(e) and 4(f) illustrate how three factors affect the response *k*. The generated ACNs' drug release was shown to be significantly impacted by factor A, with a drop in *k* observed with an increase in polymer content. This could be because as the level of factor A increases, the *k* decreases due to an increase in particle size. The diffusion path length would increase with increasing particle size, which could lead to a reduction in the drug release rate. The impact of the factor B was noteworthy, as evidenced by the observation of reduced drug release with an increase in concentration. The primary emulsion's outer volatile organic phase (chloroform) may interact more with the external aqueous phase as surfactant concentration rises, slowing the rate of evaporation. As a result, the ACNs with a stiffer membrane were produced, and the drug release rate was reduced [39, 48]. Factor C also had an impact, as an increase in glycerol concentration was accompanied by a decrease in *k* value. This may be the result of the viscosity increasing at higher glycerol concentrations, which may have slowed the evaporation of chloroform. The polymer membrane of the nanocapsules produced by delayed evaporation rates may have been stiffer and less porous [48], which may have slowed the pace at which drugs are released from them. All three factors' effects were determined to be significant at *p* < 0.05 (Table 3).

All LMV-ACN formulations were shown to fit the first-order kinetics of drug release, as shown by the zero- and first-order kinetic plots. The non-Fickian diffusion mechanism of drug release was determined using Higuchi's and Korsmeyer–Peppas plots.

### 3.8. Design Validation

The adjusted and predicted *R*^2^ values for each response variable were found to be close, with a difference between them of less than 0.2, suggesting that this may be explored for optimization or design space development.  Table 3 displays the results of the ANOVA. Each response's model *F* value indicates that the model was significant at *p* < 0.05. This suggests that the response surface quadratic model was suitable for elucidating the impact of the factors on the responses. Furthermore, supporting this was the negligible lack-of-fit values. These findings signified that this could be extended to further optimization.

### 3.9. Optimization

**Figure 5:**
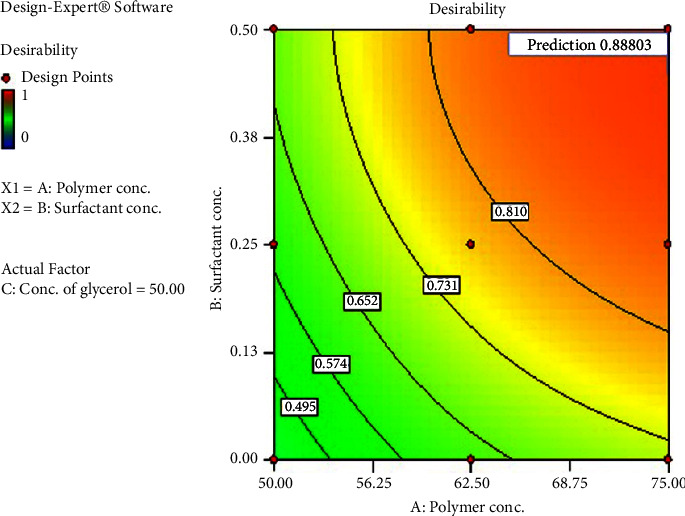
Desirability plot showing the maximum desirability for the set desirability criteria.

Out of all the alternatives the software offered for the set desire, one solution (as illustrated in Figure 5) had the highest desirability of 0.888 at a combination of factors of 75% w/w of the factor A, 0.45% w/v of the factor B, and 50% v/v of the factor C. At this combination, the values of the response factors are, as indicated by the software, 70.69% of EE, 294.99 nm of PS, and 0.099 h^−1^ of *k*. Using this ideal combination of the factors, a new formulation of LMV-ACNs was created and the characterization tests were carried out for the response variables. The results showed that the EE was 71.54%, the PS was 288.4 nm with a polydisperse index (PDI) of 0.245, the zeta potential was −24.7 mV, and the *k* was 0.095 h^−1^. These outcomes and the ones provided by the design showed a strong correlation. As a result, the optimal formulation of LMV-ACNs was thought to be this combination of the three factor levels.

### 3.10. Surface Modification

The zeta potential of the optimized LMV-ACNs was −24.7 mV. This negative zeta potential might be due to the polymer PLGA RG503H which contains free carboxylic acid groups as the end groups on it [49, 50]. This high negative zeta potential is an advantage as it can control the aggregation of ACNs in the dispersion and can improve the physical stability.

The literature states that nanoparticles with high negative zeta potential can be easily opsonized and cleared from the blood to reach RES-rich organs such as the liver [51]. So, the optimized ACNs of LMV were further treated with SLS in order to increase their negative zeta potential. After treatment with SLS, the zeta potential of LMV-ACNs was observed to be increased to a maximum zeta potential of −41.6 mV at a 6 mM concentration of SLS. Hence, with this zeta potential, the ACNs were assumed to have more targetability to reach the liver, which had to be confirmed by *in vivo* biodistribution studies.

### 3.11. *In Vitro* Cytotoxicity Study

**Figure 6:**
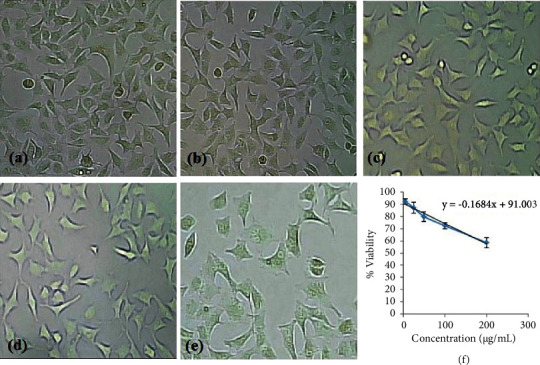
MTT assay images showing (a)–(e) the intensity of cells at different concentrations of LMV-ACNs ranging 5–200 *μ*g/mL and (f) plot of concentration versus % viability. (^*∗*^The data presented were the mean with standard deviation as the error bars for three observations).

This study was conducted on the optimized LMV-ACNs that were prepared with PLGA RG503H. The reason behind the use of the HeLa cell lines is that these cells are resistant to cell death by natural apoptosis, and they are destroyed only due to the cytotoxicity of the test substances. So, these cell lines can give accurate results of cytotoxicity by the test substance. There were five concentrations employed, ranging from 5 to 200 *μ*g/mL. Figures 6(a)through 6(e) depict the images of the cells following the therapy. A viability plot (Figure 6(f)) was created from the obtained % viability at these concentrations by creating a linear regression equation. The concentration of the test substance needed to prevent the development of 50% of cells is known as half the maximum inhibitory concentration, or IC_50_ [52]. It was discovered that LMV-ACNs had an IC_50_ of 243.5 ± 11.8 *μ*g/mL. This high IC_50_ (a 300 mg adult dose produces a maximum plasma concentration of 1.4 *μ*g/mL; hence, this IC_50_ is approximately 173.5 times the dose) value indicates that both LMV and PLGA RG503H in the LMV-ACNs are not toxic and relatively safe.

### 3.12. *In Vivo* Biodistribution/Bioavailability Studies

**Figure 7:**
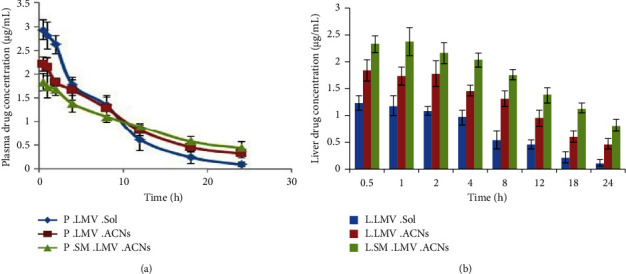
(a) Plot of time versus plasma drug concentration of LMV; (b) plot of time versus liver drug concentration of LMV. (^*∗*^The data presented were the mean of three observations, and the comparison was made at a significance limit of *p* < 0.05).

*In vivo* biodistribution/bioavailability studies were performed for pure LMV solution and optimized LMV-ACNs and surface-modified LMV-ACNs in rats. From the HPLC results, the LMV was found to be eluted at 2.38 min. and the Nelfinavir (IS) was eluted at 3.57 min. The ratios of the peak areas of the LMV and the IS were equated against the previously developed calibration curve to quantify the plasma concentration of the LMV. The obtained data of plasma and liver drug concentrations are illustrated in Figures 7(a) and 7(b), respectively. These data were subjected to noncompartmental analysis to find various pharmacokinetic properties so as to understand the impact of formulation of ACNs and their surface modification on plasma and the hepatic bioavailability of LMV. The results are shown in Table 4. In plasma, steady-state volume of distribution (*V*_ss_) and elimination half-life (*t*_1/2_) increased when formulating LMV into ACNs which indicates that LMV distributes more into the body as the nanoparticles can diffuse into various tissues. Whereas for the data obtained from the liver tissue, the *V*_ss_ was found to decrease from 0.58 L/kg of LMV solution to 0.23 L/kg of LMV-ACNs. This indicated that more concentration of the drug was confined in the liver since only fewer amounts were distributed out of the liver. This could be attributed to the surface hydrophobicity [53, 54] and negative zeta potential of the LMV-ACNs as they were made of PLGA that might induce their phagocytosis and make them more available to the liver [12, 50]. This could also be further justified by the observed hepatic AUC, which increased to 32.94 *μ*g/mL *∗* h for the LMV-ACNs compared to 13.78 *μ*g/mL *∗* h for pure LMV solution. So, it is evident that LMV-ACNs prepared from PLGA make the LMV more available in the liver where it is actually needed. Still, a further significant (*p* < 0.05) increase in hepatic AUC (66.7%) and decrease in *V*_*d*_ (64.3%) were observed upon surface modification of LMV-ACNs with SLS. This might be due to the increased negative zeta potential which could improve the phagocytosis of nanoparticles into RES-rich organs such as the liver. This was further supported by a significant increase (*p* < 0.05) in hepatic mean residence time (MRT) (34.7%) and a decrease in clearance (19.7%) upon surface modification of LMV-ACNs.

These observed results are justified by the work reported by Nag et al. [55] regarding the passive targeting ability of the PLGA nanoparticles. These authors developed PLGA nanoparticles for loading tannic acid (TA) and vitamin E for the treatment of alcoholic liver damage (ALD). The prepared nanoparticles were found to have a zeta potential of −21.2 mV. The *in vivo* and histopathology results revealed that recovery of the liver was highest in the animals treated with the PLGA-TA-E nanoparticles than those treated with plain TA and plain vitamin E. The authors ascribed this result to the improved delivery of the TA and vitamin E to the liver by the nanoparticles owing to their hydrophobic nature and surface charge. In one more study reported by Zhang et al. [56], Cholesterol-based nanoparticles were developed for loading miRNA to treat ALD. Besides, the miRNA was paired with polyethyleneimine (PEI) and made into nanoparticles. *In vivo* biodistribution and histopathology studies revealed greater accumulation of the RNA from the cholesterol-based nanoparticles than the PEI-paired RNA. The authors attributed this observation to the surface hydrophobicity of the nanoparticles owing to the presence of cholesterol. Few other similar reports regarding the passive liver targeting ability of nanoparticles are reviewed by Warner et al. [57].

These recent literature reports suggested that the proposed mechanism of liver targeting in this work is justified. Besides the charge, the surface hydrophobicity of the PLGA nanoparticles to a greater is responsible for the passive liver targeting. Opsonins in the blood easily detect and attack the hydrophobic particles that are administered into the blood through the IV route. The opsonized nanoparticles can be readily engulfed by the reticuloendothelial system (RES) rich organs such as liver and spleen [58, 59]. This mechanism of phagocytosis is even more prominent for the nanoparticles with a negative charge and with a size of above 200 nm [57]. These possible mechanisms could be responsible for the greater accumulation of the LMV from the PLGA nanoparticles developed in this work.

## 4. Conclusion

The current research work was executed out with the objective of achieving liver targetability by developing biodegradable nanocapsules for delivering LMV. The experiment was designed successfully with 3^3^full-factorial design. All three selected formulation parameters were observed to have a quadratic effect on the three responses. This further proceeded to graphical optimization. The optimized formulation was obtained as ACNs containing PLGA at 75% w/w, Pluronic-F68 at 0.45% w/v, and glycerol in the external phase at 50.00% v/v. The optimized ACNs were further coated with SLS so as to increase the zeta potential. The *in vivo* biodistribution studies indicated that the optimized ACNs increased the hepatic bioavailability of LMV by 139% when compared to pure LMV. Further, surface modification of the optimized ACNs resulted in an increase in the hepatic bioavailability of LMV by 66.7% when compared to the optimized ACNs. These findings designated that the hepatic targetability was accomplished by developing the ACNs and was further increased by surface modification, thus demonstrating the successful achievement of the study objectives. Hence, this ACN formulation can be extended to drugs like LMV in achieving liver-specific delivery to enhance their therapeutic outcomes.

## Figures and Tables

**Table 1:** Description of the three formulation factors and their levels taken in this work.

Factor	Description	Level		
		−1	0	+1
A	Polymer concentration in final weight of nanocapsules (% w/w)	50	62.5	75
B	Surfactant concentration in secondary emulsion (% w/v)	0	0.25	0.5
C	Concentration of glycerol in external phase (% v/v)	0	25	50

**Table 2:** Combinations of the formulation factors according to the selected factorial design and the observed results^*∗*^ of characterization studies of LAM-ACNs.

Formulation code	Factor level			EE^a^ (%)	Particle size (nm)	Zeta potential (mV)	*k* ^c^ (h^−1^)
	A	B	C				
F1	−1	−1	−1	21.59 ± 3.18	305.2 ± 3.4	−24.6 ± 1.5	0.243 ± 0.02
F2	−1	0	−1	32.14 ± 2.54	289.3 ± 2.5	−26.9 ± 1.7	0.224 ± 0.05
F3	−1	+1	−1	28.42 ± 2.67	274.3 ± 1.9	−25.7 ± 0.9	0.206 ± 0.03
F4	−1	−1	0	32.68 ± 3.22	302.1 ± 5.2	−26.3 ± 2.1	0.218 ± 0.03
F5	−1	0	0	48.29 ± 1.96	286.5 ± 4.2	−22.9 ± 2.3	0.186 ± 0.04
F6	−1	+1	0	42.06 ± 4.06	272.3 ± 2.6	−25.3 ± 1.4	0.165 ± 0.02
F7	−1	−1	+1	44.52 ± 3.28	268.3 ± 2.1	−24.3 ± 1.8	0.196 ± 0.06
F8	−1	0	+1	54.38 ± 4.25	242.5 ± 3.2	−24.9 ± 0.8	0.174 ± 0.03
F9	−1	+1	+1	52.29 ± 2.98	231.3 ± 1.8	−26.2 ± 1.5	0.147 ± 0.04
F10	0	−1	−1	38.96 ± 2.17	324.8 ± 2.7	−25.6 ± 2.3	0.206 ± 0.03
F11	0	0	−1	43.58 ± 3.26	318.2 ± 5.7	−23.7 ± 1.7	0.197 ± 0.01
F12	0	+1	−1	42.19 ± 3.09	304.3 ± 3.1	−24.3 ± 1.1	0.165 ± 0.03
F13	0	−1	0	46.36 ± 4.21	322.6 ± 3.6	−22.9 ± 3.1	0.186 ± 0.02
F14	0	0	0	57.47 ± 3.84	313.9 ± 2.3	−24.1 ± 2.4	0.178 ± 0.06
F14	0	0	0	56.39 ± 2.61	310.6 ± 1.4	−25.3 ± 1.9	0.169 ± 0.04
F15	0	+1	0	52.62 ± 2.46	299.6 ± 2.8	−23.9 ± 1.8	0.154 ± 0.02
F16	0	−1	+1	59.72 ± 4.22	315.9 ± 3.5	−24.9 ± 2.1	0.143 ± 0.03
F17	0	0	+1	69.84 ± 1.96	302.5 ± 2.2	−25.4 ± 1.3	0.126 ± 0.05
F18	0	+1	+1	66.92 ± 2.05	294.1 ± 4.7	−23.7 ± 3.2	0.112 ± 0.02
F19	+1	−1	−1	36.85 ± 2.31	341.2 ± 4.3	−28.1 ± 2.6	0.175 ± 0.04
F20	+1	0	−1	42.52 ± 1.87	320.5 ± 3.9	−27.3 ± 2.9	0.162 ± 0.03
F21	+1	+1	−1	40.36 ± 3.04	311.6 ± 1.5	−25.2 ± 1.6	0.148 ± 0.04
F22	+1	−1	0	52.39 ± 2.12	336.1 ± 1.9	−26.7 ± 1.2	0.159 ± 0.05
F23	+1	0	0	59.86 ± 3.28	323.5 ± 2.4	−24.6 ± 1.9	0.153 ± 0.02
F24	+1	+1	0	53.58 ± 2.31	308.1 ± 1.5	−22.7 ± 0.7	0.138 ± 0.02
F25	+1	−1	+1	63.93 ± 1.86	322.5 ± 3.1	−26.2 ± 2.2	0.121 ± 0.04
F26	+1	0	+1	73.41 ± 2.13	302.4 ± 2.5	−26.7 ± 2.1	0.108 ± 0.03
F27	+1	+1	+1	69.35 ± 2.54	286.9 ± 2.3	−25.8 ± 2.7	0.094 ± 0.02

^*∗*^The results were expressed as average ± standard deviation for *n* = 3.

**Table 3:** Results of ANOVA for the quadratic model for three selected response variables.

S. no.	Response	Source	SS^a^	Df^b^	MSS^c^	*F*value	*p*value	Inference^d^
1	EE^h^ (%)	Model	4617.96	9	513.11	174.90	<0.0001	Significant
		A^e^	1025.74	1	1025.74	349.63	<0.0001	Significant
		B^f^	143.31	1	143.31	48.85	<0.0001	Significant
		C^g^	2881.67	1	2881.67	982.24	<0.0001	Significant
		AB	16.01	1	16.01	5.46	0.0313	Significant
		AC	26.76	1	26.76	9.12	0.0074	Significant
		BC	3.88	1	3.88	1.32	0.2654	Not significant
		A^2^	214.65	1	214.65	73.17	<0.0001	Significant
		B^2^	264.47	1	264.47	90.15	<0.0001	Significant
		C^2^	1.08	1	1.08	0.37	0.5522	Not significant
		Residual	52.81	18	2.93			
		Lack of fit	52.22	17	3.07	5.27	0.3315	Not significant

2	Particle size (nm)	Model	16802.45	9	1866.94	38.11	<0.0001	Significant
		A	8064.50	1	8064.50	164.60	<0.0001	Significant
		B	3464.58	1	3464.58	74.43	<0.0001	Significant
		C	2762.72	1	2762.72	56.39	<0.0001	Significant
		AB	1.69	1	1.69	0.034	0.8548	Not significant
		AC	354.25	1	354.25	7.23	0.0150	Significant
		BC	14.96	1	14.96	0.31	0.5873	Not significant
		A^2^	1312.04	1	1312.04	26.78	<0.0001	Significant
		B^2^	19.61	1	19.61	0.40	0.5349	Not significant
		C^2^	533.27	1	533.27	10.88	0.0040	Significant
		Residual	881.88	18	48.99			
		Lack of fit	876.43	17	51.55	9.47	0.2508	Not significant

3	*k* ^i^ (h^−1^)	Model	0.035	9	3.84 × 10^−3^	63.80	<0.0001	Significant
		A	0.014	1	0.014	231.81	<0.0001	Significant
		B	5.62 × 10^−3^	1	5.62 × 10^−3^	93.39	<0.0001	Significant
		C	0.014	1	0.014	235.53	<0.0001	Significant
		AB	3.41 × 10^−4^	1	3.41 × 10^−4^	5.67	0.0285	Significant
		AC	3.00 × 10^−6^	1	3.00 × 10^−6^	0.05	0.8258	Not significant
		BC	3.33 × 10^−7^	1	3.33 × 10^−7^	0.0055	0.9415	Not significant
		A^2^	1.36 × 10^−4^	1	1.36 × 10^−4^	2.27	0.1493	Not significant
		B^2^	3.10 × 10^−5^	1	3.10 × 10^−5^	0.52	0.4819	Not significant
		C^2^	3.16 × 10^−4^	1	3.16 × 10^−4^	5.25	0.0342	Significant
		Residual	1.08 × 10^−3^	18	6.13 × 10^−5^			
		Lack of fit	1.04 × 10^−3^	17	4.05 × 10^−5^	1.51	0.5724	Not significant

*Note*. ^a^Sum of squares; ^b^degrees of freedom; ^c^mean sum of squares; ^d^*p* value less than 0.05 indicates model terms are significant; ^e^polymer concentration (%w/w); ^f^surfactant concentration in secondary emulsion (%w/v); ^g^concentration of glycerol in external phase (%v/v); ^h^entrapment efficiency; ^i^release rate constant.

**Table 4:** Results showing various pharmacokinetic parameters after noncompartmental analysis of plasma and liver data obtained from in vivo biodistribution studies of LMV-ACNs.

S. no.	Pharmacokinetic parameter	Plasma			Liver		
		LMV Solution	LMV-PLGA ACNs	Surface-modified LMV-PLGA ACNs	LMV Solution	LMV-PLGA ACNs	Surface-modified LMV-PLGA ACNs
1	*t*_1/2_ (h)	4.23 ± 0.48	8.13 ± 0.89	11.14 ± 1.24	6.58 ± 1.37	11.56 ± 0.77	15.68 ± 0.90
2	AUC (*μ*g/mL *∗* h)	24.34 ± 4.34	27.78 ± 4.94	30.23 ± 5.76	13.78 ± 3.48	32.94 ± 5.12	54.91 ± 6.68
3	MRT (h)	6.53 ± 0.95	11.64 ± 1.29	16.21 ± 2.20	9.53 ± 1.82	16.52 ± 1.19	22.25 ± 1.52
4	V_ss_ (L/kg)	0.32 ± 0.06	0.28 ± 0.05	0.26 ± 0.05	0.58 ± 0.15	0.23 ± 0.04	0.14 ± 0.02
5	Cl_T_ (L/kg/h)	2.05 ± 0.07	3.21 ± 0.22	4.10 ± 0.23	5.32 ± 0.34	3.85 ± 0.32	3.09 ± 0.17

*t *_1/2_: elimination half-life; AUC: area under the time-plasma drug concentration curve; MRT: mean residence time; V_ss_: steady state volume of distribution; Cl_T_: total body clearance. (^*∗*^The data presented were the mean of three observations, and comparison was made at a significance limit of *p* < 0.05)

## Data Availability

All the necessary data sufficient to understand the work are presented in this manuscript.
